# Dynamic Notch Signaling Specifies Each Cell Fate in *Drosophila* Spermathecal Lineage

**DOI:** 10.1534/g3.117.040212

**Published:** 2017-03-03

**Authors:** Wei Shen, Jianjun Sun

**Affiliations:** * Department of Physiology and Neurobiology, University of Connecticut, Storrs, Connecticut 06269; † Institute for Systems Genomics, University of Connecticut, Storrs, Connecticut 06269

**Keywords:** class-III secretory gland, spermathecae, Notch signaling, binary cell fate determination, Cut

## Abstract

Spermathecae are glandular organs in the insect female reproductive tract that play essential roles in insect reproduction; however, the molecular mechanism involved in their development is largely unknown. *Drosophila* spermathecae consist of class-III secretory units, in which each secretory cell (SC) discharges its products to the central lumen through an end-apparatus and a canal. Secretory unit formation in *Drosophila* spermathecae utilizes a fixed cell lineage, in which each secretory unit precursor (SUP) divides to produce one pIIb cell and one pIIa cell. The former differentiates into an apical cell (AC), whereas the latter divides again to produce an SC and a basal cell (BC). It is unclear how each cell acquires its identity and contributes to secretory unit formation. Here, we demonstrate that Notch signaling is required and sufficient for the specification of lumen epithelial precursors (LEPs; *vs.* SUPs), pIIb (*vs.* pIIa), and SCs (*vs.* BCs) sequentially. To our surprise, Notch activation in LEPs and SCs apparently utilizes different ligand mechanisms. In addition, Notch signaling both suppresses and activates transcription factors Hindsight (Hnt) and Cut during spermathecal lineage specification, supporting the notion that Notch signaling can have opposite biological outcomes in different cellular environments. Furthermore, LEP-derived epithelial cells (ECs) and ACs show distinct cellular morphology and are essential for securing secretory units to the epithelial lumen. Our work demonstrates, for the first time, the dynamic role of Notch signaling in binary cell fate determination in *Drosophila* spermathecae and the role of ECs and ACs in secretory unit formation.

Spermathecae are sperm-storage organs found in the female reproductive tract of many insect species, and they are important for reproduction and cryptic female choice (Eberhard 1996; Pitnick *et al.* 1999; Manier *et al.* 2010). Studies in *Drosophila* have shown that glandular secretions from spermathecae and parovaria act to attract, nourish, and protect sperm by creating an appropriate environment (Filosi and Perotti 1975; Allen and Spradling 2008; Prokupek *et al.* 2008, 2009; Schnakenberg *et al.* 2011; Sun and Spradling 2013). This is likely true in other insect species (Shaw *et al.* 2014). In addition, glandular secretions from spermathecae and parovaria regulate ovulation and egg laying (Schnakenberg *et al.* 2011; Sun and Spradling 2013; Cattenoz *et al.* 2016). Although the exact identities of the secreted products regulating sperm and ovulation are unknown, it is clear that secretions through the canonical protein secretory pathway are required for sperm storage but not ovulation (Sun and Spradling 2013). Despite recent progress on the physiology of spermatheca secretion, the molecular mechanisms involved in spermathecal gland formation are largely unknown.

Spermathecae in *Drosophila melanogaster* are a pair of mushroom-shaped organs with a head capsule connected to the reproductive tract by an epithelial duct (Filosi and Perotti 1975). The head capsule contains a brown-pigmented cuticular lumen surrounded by a layer of ECs and polyploid SCs. Ultrastructural investigations showed that each SC has an apical extracellular reservoir (named the end-apparatus), which is connected to the central lumen by a cuticular canal (Filosi and Perotti 1975; Allen and Spradling 2008; Mayhew and Merritt 2013). SCs discharge their secretions to the central lumen through the end-apparatus and the canal, all of which make up the secretory unit. Similar secretory units are also found in *Drosophila* parovaria (Allen and Spradling 2008) and spermathecae of cockroaches (Gupta and Smith 1969), mealworms (Happ and Happ 1977), *Rhodnius* (Lococo and Huebner 1980), springtails (Dallai *et al.* 2008), and mosquitoes (Pascini *et al.* 2012, 2013; Laghezza Masci *et al.* 2015). This type of secretory units is also found in epidermal glands, which are categorized into three classes according to the morphology of the SC and the way of discharge of the secretion (Noirot and Quennedey 1974). In class-I and class-II glands, SCs discharge their secretions directly across the cuticle and indirectly through epidermal cells, respectively. In contrast, class-III glands discharge their secretion through a complex, extracellular end-apparatus and a cuticular canal, which are constructed by one or more supporting cells. (Noirot and Quennedey 1974; Quennedey 1998).

Primordia of spermathecae and parovaria are mapped to specific segments in the *Drosophila* genital imaginal disc, which gives rise to the female lower reproductive tract during pupae development (Keisman *et al.* 2001). The sex determination cascade activates the runt-domain transcription factor Lozenge (Lz) in these primordial cells, which is essential for gland formation (Anderson 1945; Chatterjee *et al.* 2011; Sun and Spradling 2012). NR5A-family nuclear receptor Hr39 regulates the proliferation, survival, and protrusion of these primordial cells during early pupae development; Hr39 expression is likely regulated by the transcription factor Glial cell missing (Allen and Spradling 2008; Sun and Spradling 2012; Cattenoz *et al.* 2016). Precursor cells except those at the middle region of the spermathecal head continue to express Lz and differentiate into epithelia cells (ECs) to form adult spermathecal lumen and duct. In contrast, precursor cells at the middle region of the spermathecal head divide to give rise to LEPs and SUPs by 26 hr after puparium formation (26 hr APF; Figure 1A and Sun and Spradling 2012). LEPs continue to express Lz and differentiate into lumen ECs, whereas SUPs downregulate Lz expression, activate transcription factor Hnt, and divide stereotypically into three-cell secretory units, including an AC, an SC, and a BC (Figure 1A and Sun and Spradling 2012, 2013). The three cells in the secretory unit wrap around each other to form a concentric ring during secretory unit morphogenesis, and both ACs and BCs disappear in adult secretory units, likely through programmed cell death (Sun and Spradling 2012; Mayhew and Merritt 2013). However, it is unclear how the three cells in the secretory unit acquire their identity and contribute to the formation of the sophisticated secretory unit of adult spermathecae and parovaria.

**Figure 1:**
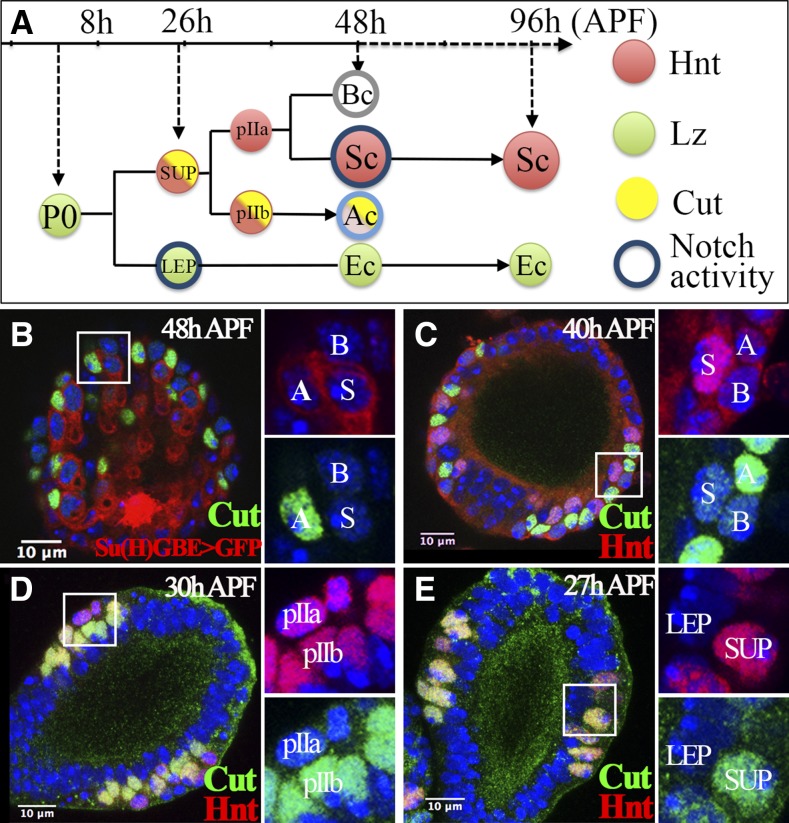
Cut expression in the spermathecal lineage. (A) Diagram depicting the spermathecal lineage during *Drosophila* pupal development. Hnt, Lz, and Cut expression are labeled in red, green, and yellow, respectively. Notch activity is indicated by the blue circle. A light blue circle was used in the AC to reflect the lower expression of Notch activity reporter at 48 hr APF. (B) Cut expression (green) in spermathecae at 48 hr APF. Notch activity is indicated by *Su(H)GBE-Gal4* driving expression of *UAS-GFP* (*Su(H)GBE* > *GFP*; shown in red). Enlarged pictures of the squared area with two channels are shown in the right two panels. The AC, BC, and SC are marked by A, B, and S, respectively. DNA staining with DAPI is shown in blue in all figures. (C–E) Expression of Cut (green) and Hnt (red) in spermathecae at 40 (C), 30 (D), and 27 hr (E) APF. All figures depict the spermathecal heads oriented with distal head (vault) pointed upwards and the duct pointed downward. AC, apical cell; APF, after puparium formation; BC, basal cell; DAPI, 4’,6-diamidino-2-phenylindole; Ec, epithelial cell; GFP, green fluorescent protein; Hnt, Hindsight; LEP, lumen epithelial precursors; Lz, Lozenge; SC, secretory cell; Su(H), Suppressor of Hairless; SUP, secretory unit precursors.

Notch signaling is an evolutionarily conserved signaling pathway, which can be activated ligand-dependently or ligand-independently (Palmer and Deng 2015). Notch signaling has been implicated in binary cell fate determination in many organs with fixed cell lineage (Artavanis-Tsakonas *et al.* 1999; Lai and Orgogozo 2004; Schweisguth 2015). The detection of Notch activity in LEPs and SCs (Figure 1A and Sun and Spradling 2013) led us to investigate its role in secretory unit formation in *Drosophila* spermathecae. We first identified the homeobox transcription factor Cut expressed in SUPs and restricted to pIIb/ACs. With the expression of Lz, Hnt, and Cut marking each cell fate in the spermathecal lineage, we showed that canonical Notch signaling is required and sufficient for the specification of LEP, pIIb, and SC fate in the spermathecal lineage. Notch ligand Serrate (Ser) seems not to be involved in Notch signaling in the spermathecal lineage. Interestingly, Notch ligand Delta (Dl) is required for LEP specification, but not for SC fate. By manipulating Notch signaling to transform cell fate, we demonstrate that ECs and ACs are essential to secure the secretory unit to the epithelial lumen and that each cell in the spermathecal lineage shows distinct cellular morphology consistent with their biological functions.

## Materials and Methods

### Drosophila genetics

Flies were reared on standard cornmeal–molasses food at 25°, unless otherwise indicated. *Su(H)GBE-lacZ* (Furriols and Bray 2001) and *Su(H)GBE-Gal4* (Zeng *et al.* 2010) were used to monitor Notch activity. *UAS-mCD8:GFP*, *lz-Gal4* (Crew *et al.* 1997), *51B02-Gal4* (Sun and Spradling 2013), and *hsFLP; act < CD2 < Gal4, UAS-GFP* (flip-out Gal4 system; Sun and Deng 2005) flies were crossed to UAS-X lines for overexpression in specific cells. The following UAS-X lines were used: *UAS-N^RNAi^* (a gift from S. Bray), *UAS-Su(H)DN* (Mukherjee *et al.* 2011), *UAS-Dl^RNAi^* (B34322 and B28032; Bloomington *Drosophila Stock Center*, BDSC), *UAS-NICD* (BDSC), *UAS-Ser^RNAi^* (V108348 and V27172; Vienna *Drosophila* Resource Center), and *UAS-eGFP* (BDSC).

### Pupae collection and clone analysis

Pupae collection and dissection were similar to previously described methods (Sun and Spradling 2012). In short, white prepupae (designated as 0 hr APF) were collected over a 30-min window into a new food vial, sexed according to gonadal size, and aged to the desired pupal stage for heat shock treatment or dissection. For flip-out clone induction, pupae were heat shocked in a 37° water bath for 10–15 min, which is optimal to generate single-cell clones. Images were taken for each clone, and the cell identity of each clone was analyzed according to the molecular and morphological markers. Clones with only ECs were not included in the analysis.

### Immunocytochemistry

Antibody staining was performed as previously described (Sun and Spradling 2012). In short, the entire genital disc attached to the cuticle was fixed in 4% electron microscopy-grade paraformaldehyde for 15 min and blocked in PBTG (PBS + 0.2% Triton-X 100 + 2% normal goat serum + 0.5% BSA) before being subjected to primary and secondary antibody staining. The following primary antibodies against transcription factors were obtained from the Developmental Studies Hybridoma Bank (DSHB) and used for the antibody screen: mouse anti-EYA (1:10), anti-Cut (1:15), anti-Glass (1:15), anti-Abrupt (1:15), anti-En (1:15), anti-Pros (1:15), anti-Yan (1:15), and anti-Enabled (1:15). In addition, we also used the following primary antibodies: rabbit anti-GFP (1:2000; Life Technologies, Carlsbad, CA), and mouse anti-Lz (1:15), anti-Hnt (1:150), and anti-Arm (1:40) from the DSHB. We used the following secondary antibodies: Alexa-568 goat anti-mouse (1:1000; Life Technologies) and Alexa-488 goat anti-rabbit (1:1000; Life Technologies). Nuclei were stained with DAPI (Sigma-Aldrich, St. Louis, MO). All images were captured with a Leica SP8 confocal microscope, processed with ImageJ or Imaris 3D (Bitplane, Zurich, Switzerland) software, and assembled in Adobe Photoshop (Adobe Systems, San Jose, CA).

### Data availability

All strains used in this study are available upon request. Supplemental Material, File S1 and File S2 are supplemental movies showing serial optical sectioning of N-knockdown clones with four and eight BCs, respectively. File S3 contains supplemental figure legends. 

## Results

### Homeobox transcription factor Cut is restricted to ACs during secretory unit formation

To investigate secretory unit formation, we performed a small-scale antibody screen to identify transcription factors expressed in specific cell types in the spermathecal lineage. Eight antibodies were tested (see *Materials and Methods*), and only one against homeobox transcription factor Cut showed a unique expression pattern in the spermathecal head at 48 hr APF (Figure 1B). Expression and function of Cut has been characterized in multiple developmental organs including wing imaginal discs, sensory organs, ovarian follicle cells, and tracheal cells (Blochlinger *et al.* 1991; de Celis and Bray 1997; Sun and Deng 2005; Pitsouli and Perrimon 2013), but not in spermathecae. Although the expression of Cut was sporadic in spermathecae at 48 hr APF, it was always located in a single cell closely associated with an SC that had high Notch activity (Figure 1B). In addition, *Cut^+^* cells had elongated nuclei and displayed faint Notch activity signals (Figure 1B), hallmarks of ACs (Sun and Spradling 2012, 2013). Thus, Cut is expressed in ACs at 48 hr APF.

To investigate the timing of Cut expression in the spermathecal lineage, we first examined spermathecae at 40 hr APF, when cell divisions have completed and three-cell secretory units have formed. At this time, Cut was already expressed in ACs that showed faint Hnt expression and were juxtaposed with Hnt^+^ SCs (Figure 1C). At 30 hr APF, when SUPs start to divide, Cut already showed differential expression in pIIb but not in pIIa cells (Figure 1D); pIIb cells give rise to ACs (Figure 1A). In contrast, Hnt was equally expressed in pIIb and pIIa cells. This is the first visualization of pIIa and pIIb cells, which is predicted according to our previous lineage analysis. In addition, we noticed that pIIb cells were localized more apically toward ECs that surround the central lumen, whereas pIIa cells were localized more basally away from the lumen.

We were surprised that both Hnt and Cut were expressed in pIIb cells, as Hnt antagonizes Cut expression in follicle cells during oogenesis (Sun and Deng 2007). To determine whether Cut is also expressed in SUPs, where Hnt is expressed, we examined spermathecae at 26–28 hr APF. Indeed, SUPs expressed Cut along with Hnt as early as 26 hr APF; in contrast, Cut was not expressed in LEPs localized to the apical side (Figure 1E). Thus, at 26 hr APF, both Hnt and Cut are expressed in SUPs; later on, Cut expression is restricted to pIIb/ACs and Hnt expression is restricted to SCs (Figure 1A; Sun and Spradling 2012). Lz is expressed in LEPs and ECs. Thus, the combination of Hnt, Cut, and Lz expression will allow us to determine the cell fate of each subtype in the spermathecal lineage.

### Notch signaling specifies LEP *vs.* SUP fate

**Figure 2:**
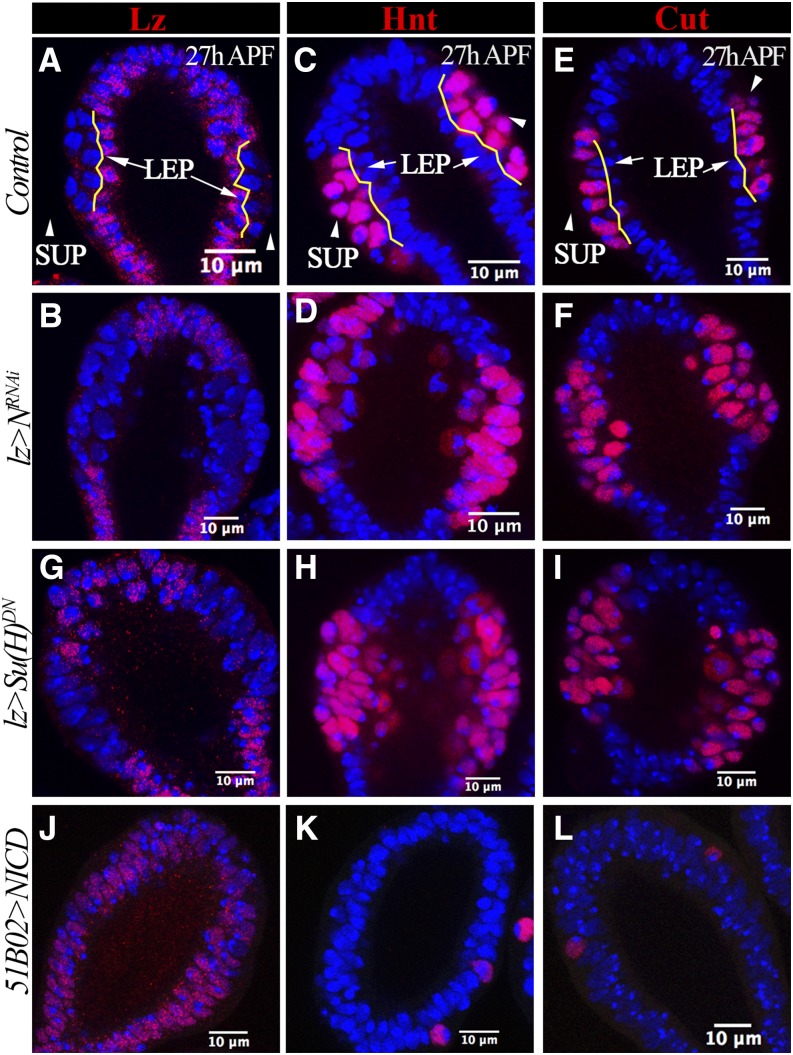
Notch is required and sufficient for LEP fate. All spermathecae are at 26–28 hr APF and at least 10 spermathecae are examined and show the same phenotype. (A–F) Expression of Lz (A and B), Hnt (C and D), and Cut (E and F) in spermathecal head. (A, C, and E) Spermathecae from the control group; (B, D, and F) depict spermathecae from *N*-knockdown pupae using *lz-Gal4 (lz* > *N^RNAi^)*. Yellow lines demarcate the apical and basal layers; LEPs in apical layers are pointed with arrows, whereas SUPs in basal layers are pointed with arrowheads. (G–I) Expression of Lz (G), Hnt (H), and Cut (I) in spermathecae with overexpression of *lz-Gal4* driving *Su(H)^DN^*. (J–L) Expression of Lz (J), Hnt (K), and Cut (L) in spermathecae with *51B02-Gal4* driving overexpression of *NICD*. APF, after puparium formation; DN, dominant negative; Hnt, Hindsight; LEP, lumen epithelial precursors; Lz, Lozenge; *N*, *Notch*; NICD, Notch intracellular domain; RNAi, RNA interference; Su(H), Suppressor of Hairless; SUP, secretory unit precursors.

Since Notch signaling is activated in LEPs at the apical side of the spermathecal head (Sun and Spradling 2013), we tested whether *Notch* (*N*) is required for the LEP fate. We used *lz-Gal4* to drive *UAS-N^RNAi^* expression in all gland precursors, and found that *N* knockdown did not disrupt the formation of the double layer in the middle region of spermathecal heads; however, expression of Lz was absent in the apical layer of *N*-knockdown *vs.* control spermathecae (Figure 2, A and B and Figure S1, A and B). As this result indicated a loss of LEP identity, we next tested whether the apical layer in *N*-knockdown spermathecae gained the SUP fate (indicated by Hnt and Cut expression). In contrast to basal expression of Hnt and Cut in control spermathecae, Hnt and Cut were expressed in all cells of the middle region of *N*-knockdown spermathecae, indicating that the apical layer did indeed gain the SUP fate (Figure 2, C–F and Figure S1, C and D). Therefore, loss of *N* led to switch LEP fate into SUP fate.

To determine whether Notch specifies LEP fate through the canonical Notch pathway, we blocked the function of the key transcription factor Suppressor of Hairless [Su(H)] by overexpressing a dominant-negative form [*Su(H)^DN^*] in gland precursors. Overexpression of *Su(H)^DN^* blocked Lz expression but induced Hnt and Cut expression in the apical layer at 26 hr APF (Figure 2, G–I), suggesting that canonical Notch signaling is essential for LEP fate.

To examine whether Notch signaling is sufficient to induce LEP fate in spermathecae, we overexpressed *Notch intracellular domain* (*NICD*) in gland precursors using *lz-Gal4*. *NICD* overexpression in gland precursors prevented spermatheca morphogenesis, arresting spermathecae at around 14 hr APF (Figure S2). To overcome this problem, we utilized *51B02-Gal4*, which is expressed in the middle region of the spermathecal head prior to expression of Hnt and Cut and continues to be expressed at 48 hr APF (Figure S3, A and B and Sun and Spradling 2013). Overexpression of *NICD* was sufficient to induce Lz expression and block Hnt and Cut expression in cells at the basal layer (Figure 2, J–L), indicating a cell fate switch from SUPs to LEPs. In sum, canonical Notch signaling is required and sufficient for the LEP fate during spermatheca development.

### Loss of LEPs leads to dissociation of the secretory unit from the central lumen

**Figure 3:**
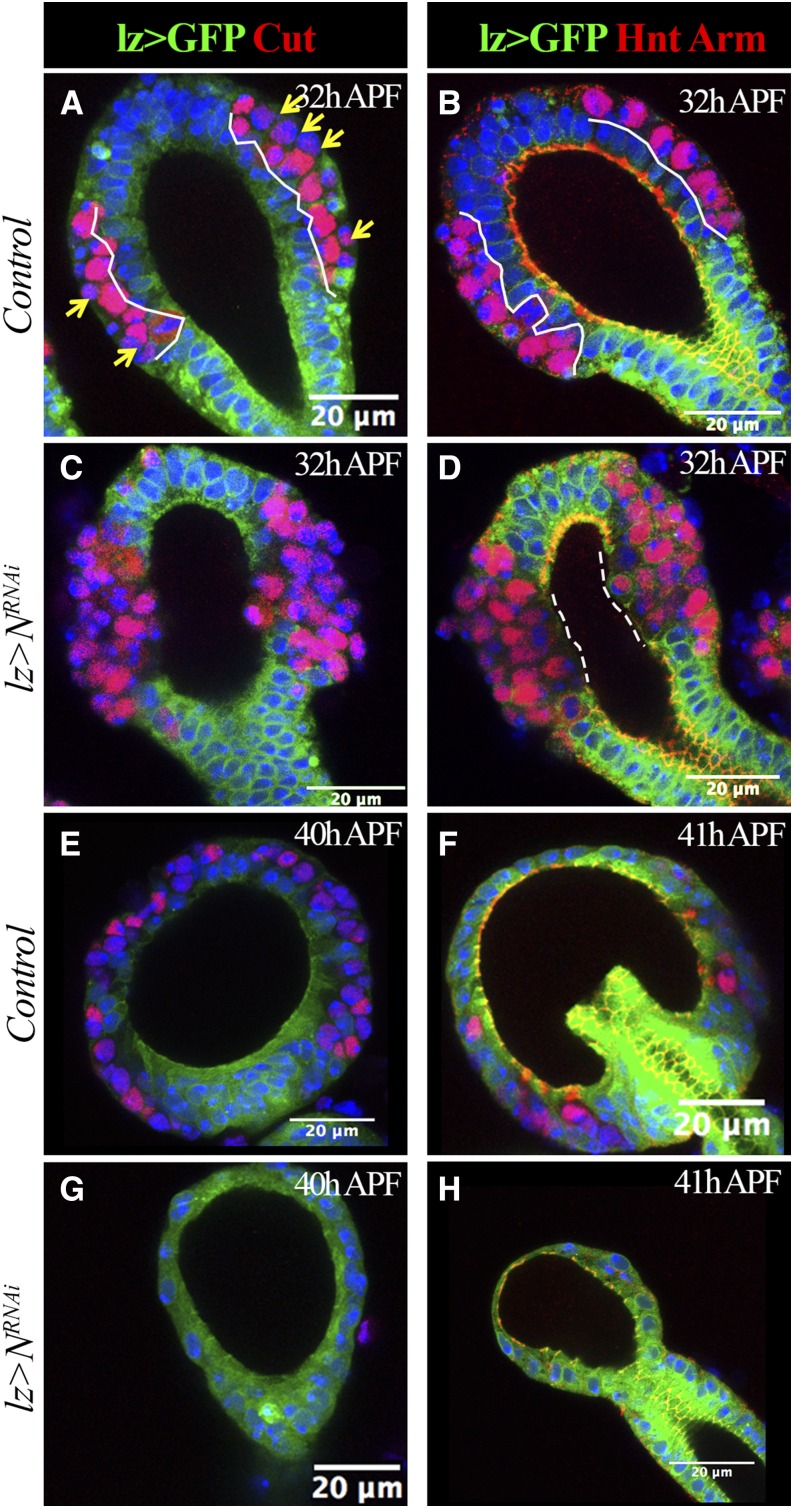
Loss of LEP causes dissociation of secretory units from the central lumen. *lz-Gal4* driving expression of *UAS-mCD8:GFP* (*lz* > *GFP*) is shown in green. Cut expression is shown in red in (A, C, E, and G). Hnt (nucleus red signal) and Arm (membrane red signal) expression are shown in (B, D, F, and H). Arm marks adherent junctions. At least 10 spermathecae are examined and shown the same pattern. (A and B) Control spermathecae at 32 hr APF. White lines demarcate the epithelial layer (GFP^+^ cells). Yellow arrows indicate pIIa cells with no or very low expression of Cut. (C and D) *N*-knockdown spermathecae at 32 hr APF. Notice the missing GFP^+^ epithelial cells in the middle region of the spermathecal head. Dashed lines demarcate the region without Arm staining. (E–H) Control (E and F) and *N*-knockdown (G and H) spermathecae at 40–41 hr APF. Only one or two Cut^+^ or Hnt^+^ cells were observed in *N*-knockdown spermathecae. APF, after puparium formation; GFP, green fluorescent protein; Hnt, Hindsight; LEP, lumen epithelial precursors; Lz, Lozenge; *N*, *Notch*; RNAi, RNA interference.

Theoretically, the transformation of LEPs into SUPs by Notch signaling inhibition will lead to more SCs in adult spermathecae. This contradicts our previous finding that inhibition of Notch signaling with *lz-Gal4* blocks SC formation in adult spermathecae and parovaria (Sun and Spradling 2013). To solve this contradiction, we examined the effect of knocking down *N* in spermathecae at later time points. At 32 hr APF, when SUPs divide to give rise to pIIa and pIIb cells, control spermathecae had a typical organization with apical ECs (*lz*^+^), middle pIIb cells (Cut^+^), and basal pIIa cells (Cut^−^; Figure 3A). Both pIIa and pIIb continue expression of Hnt (Figure 3B). In contrast, *N*-knockdown spermathecae had no apical ECs, but rather Cut^+^ (presumably pIIb cells) or Cut^−^ cells (presumably pIIa cells), both of which are Hnt positive (Figure 3, C and D). Furthermore, prominent adherent junction markers along the epithelial lumen were lost at the middle region of *N*-knockdown spermathecae (Figure 3, B and D). These data indicate that, upon *N* knockdown, LEPs indeed transform into typical SUPs that divide to give rise to pIIa and pIIb cells, consistent with the analysis at 26–28 hr APF (Figure 2).

We next examined spermathecae at 40 hr APF. To our surprise, unlike control spermathecae, the *N*-knockdown spermathecae did not have any Cut^+^ or Hnt^+^ cells at this stage (Figure 3, E–H). In fact, all the secretory units were lost in the *N*-knockdown spermathecae (Figure 3, E–H), consistent with the lack of SCs in adult *N*-knockdown spermathecae (Sun and Spradling 2013). The remaining epithelial lumen was much smaller than in the control (Figure 3, E–H). These data indicate an essential role of ECs in lumen formation and sequestering of the secretory unit to the lumen.

### Notch signaling specifies SC *vs.* BC fate

**Figure 4:**
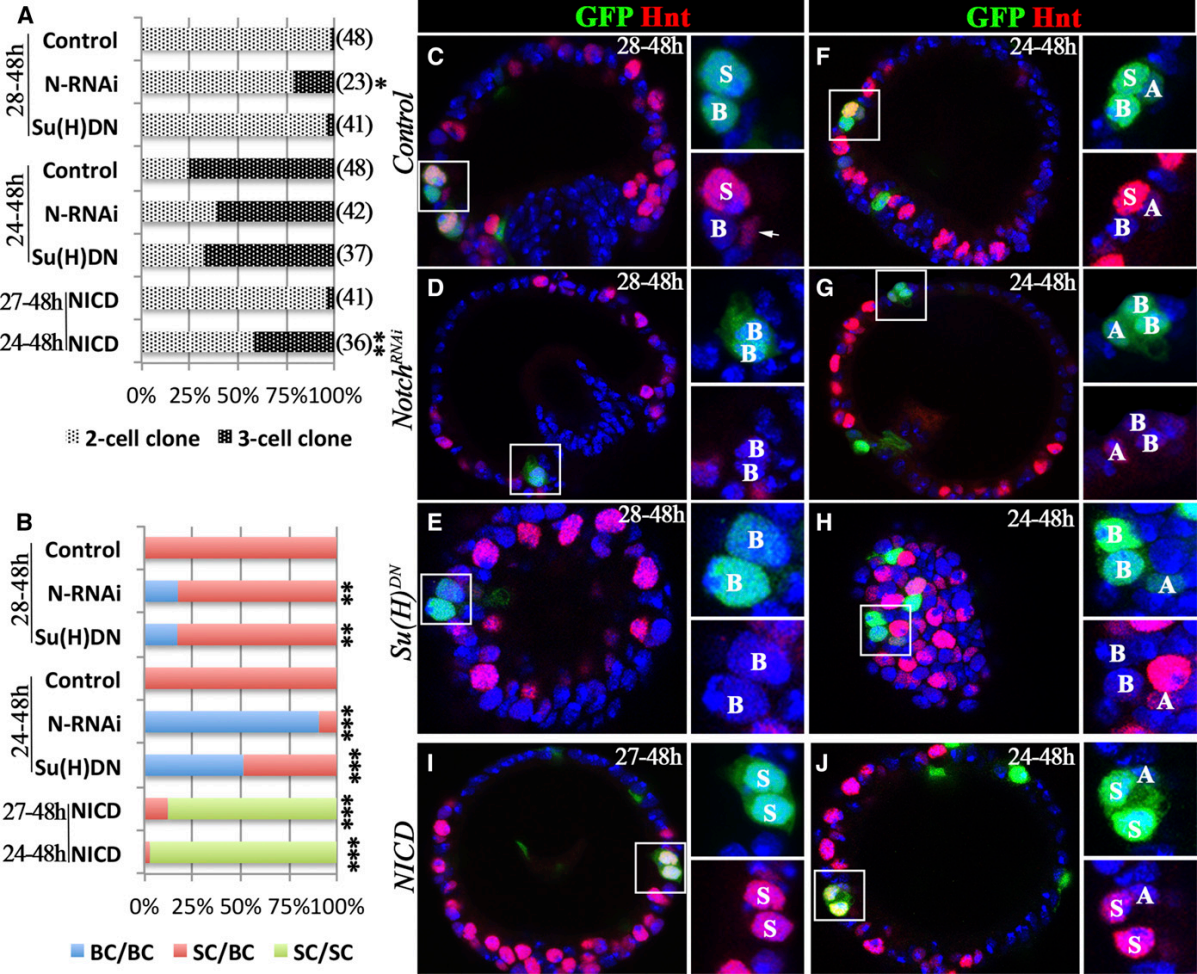
Notch signaling is required and sufficient for SC fate. Flip-out clones are marked by GFP expression (green in C–J), and Hnt expression is shown in red (C–J). (A and B) Quantification of SC-clone distribution according to clone size (A) or clone composition (B) when induced at multiple time points. The number of clones analyzed is shown in parentheses. The category in (B) only indicates the BC and SC, regardless of whether or not the clone contains the AC. Fisher’s exact test was used for assessing statistical significance (*P < 0.05, **P < 0.01, and ***P < 0.001). (C–E) Representative spermathecae with control (C), *N*-knockdown (D), or *Su(H)^DN^*-overexpressing (E) clones induced at 28 hr and observed at 48 hr APF (28–48 hr). The square areas are showed at higher magnification with only two channels in the right two subpanels. The clone cell identity is marked by A, B, or S for AC, BC, or SC, respectively. The arrow in (C) points to an AC with elongated nuclei and faint Hnt expression, which is not in the clone. (F–H) Representative spermathecae with control (F), *N*-knockdown (G), or *Su(H)^DN^*-overexpressing (H) clones induced at 24 hr and observed at 48 hr APF (24–48 hr). (I–J) Representative spermathecae with NICD-overexpressing clones induced at 27 (I) and 24 hr (J), and observed at 48 hr APF (27–48 and 24–48 hr, respectively). AC, apical cell; APF, after puparium formation; BC, basal cell; DN, dominant negative; GFP, green fluorescent protein; Hnt, Hindsight; *N*, *Notch*; NICD, Notch intracellular domain; RNAi, RNA interference; SC, secretory cell; Su(H), Suppressor of Hairless.

As Notch is also activated in SCs at 48 hr APF (Figure 1, A and B; Sun and Spradling 2013), we next probed its role in SC fate determination. To bypass the early requirement of Notch signaling in the spermathecal lineage, we utilized the flip-out Gal4 system (Pignoni and Zipursky 1997) to modify *N* expression in later development. We induced *N*-knockdown or control clones at 28 hr APF and examined them at 48 hr APF. As expected, 98% of the control SC clones were composed of two cells, invariably containing an SC (Hnt^+^) and a BC (Hnt^−^), indicating that a flip-out event occurred in a pIIa cell (Figure 4, A–C). The remaining 2% were three-cell clones composed of an AC, an SC, and a BC, indicating that a flip-out event occurred in an SUP cell. When we knocked down *N* in clones, 78% were two-cell clones; however, two out of the 18 (11%) were composed of two BCs rather than an SC and a BC (Figure 4D). Of the three-cell clones, two out of the five (40%) were composed of an AC and two BCs (Figure 4D). Both of these types of outliers indicate an SC-to-BC transformation. We confirmed the identity of the BCs by the lack of Cut staining and their unique morphology (Figure S4). In total, 17.4% of *N*-knockdown clones had an SC-to-BC transformation (Figure 4B). Similarly, 17.1% of clones with *Su(H)^DN^* overexpression showed an SC-to-BC transformation (Figure 4, B and E).

The low frequency of the SC-to-BC transformation was likely due to insufficient time to inhibit Notch signaling before cell fate specification. To test this hypothesis, we induced clones 4 hr earlier (at 24 hr APF). At this time point, 25% of control SC clones consisted of two cells (an SC and a BC) and 75% consisted of three cells (an AC, an SC, and a BC; Figure 4, A and F). With this experimental scheme, 90.5% of the clones with *N* knockdown displayed an SC-to-BC transformation: 12 out of 16 (75%) of the two-cell clones were two-BC clones and 26 out of 26 (100%) of the three-cell clones consisted of an AC and two BCs (Figure 4, B and G). Similarly, 52% of *Su(H)^DN^*-overexpressing clones had an SC-to-BC transformation (Figure 4, B and H). Altogether, these data suggest that Notch signaling is required for SC fate specification.

To determine whether Notch signaling is sufficient for SC fate, we induced *NICD* overexpression in clones at either 27 or 24 hr APF and examined them at 48 hr APF. Many of the *NICD*-overexpressing clones were composed of either two SCs (Hnt^+^; Figure 4I) or two SCs plus one AC (Figure 4J), indicating a BC-to-SC transformation. In total, 87.8 and 97.1% of *NICD*-overexpressing clones had a BC-to-SC transformation when clones were induced at 27 and 24 hr APF, respectively (Figure 4B). To determine whether *NICD* overexpression led to a full transformation of BCs into SCs, we examined these clones in adult spermathecae. Indeed, we found that the *NICD*-overexpressing clones gave rise to two developed SCs in mature spermathecae, whereas the control clones only gave rise to one SC (Figure S5). Altogether, our data suggest that Notch signaling is required and sufficient for SC fate during the SC–BC binary cell fate determination.

### Notch signaling is sufficient for pIIb fate determination

**Figure 5:**
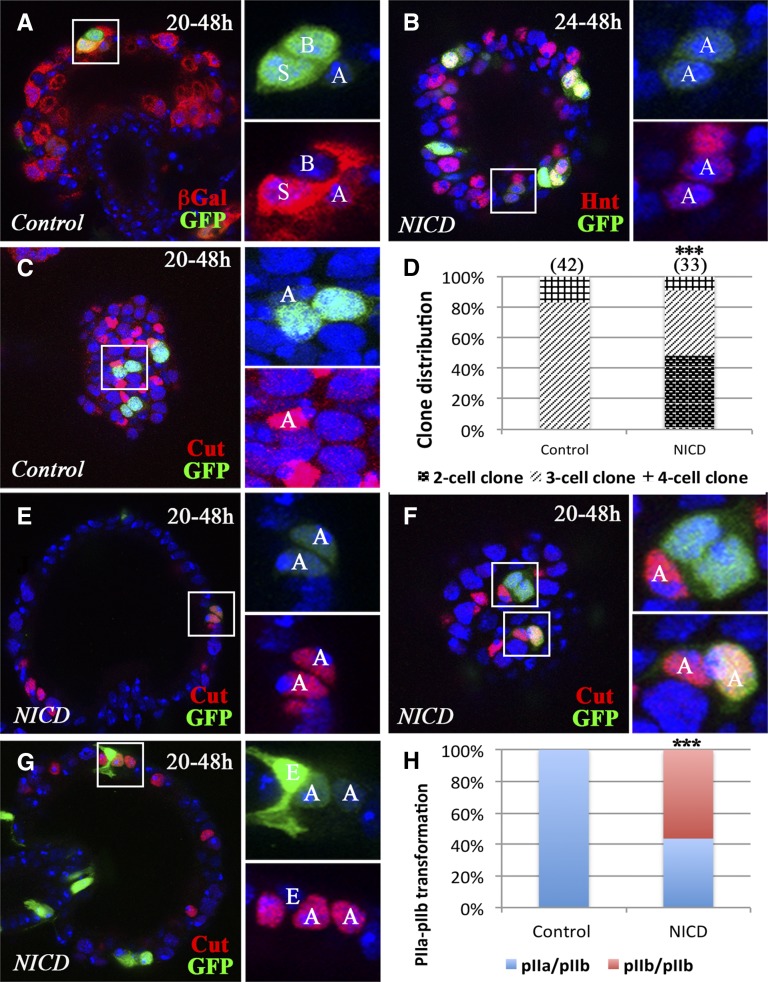
Notch signaling is sufficient for pIIb fate. Flip-out clones are marked by GFP expression (green in A–C and E–G). (A) A representative control clone induced at 20 hr APF shows weak Notch activity in the AC and strong Notch activity in the SC. βGal expression (red) from *Su(H)GBE-LacZ* reporter is used to mark Notch activity. (B) Representative *NICD*-overexpressing clones induced at 24 hr and examined at 48 hr APF. The square area is magnified in the right two panels and shows a clone with two AC-like cells with faint GFP (green) and Hnt (red) expression. (C) Representative control clones induced at 20 hr and examined at 48 hr APF. The three-cell clone in the square area shows the AC with Cut expression (red). The other two clones have the same composition. (D) Quantification of clone distribution according to clone size. Clones were induced at 20 hr APF and examined at 48 hr APF. (E–G) Representative *NICD*-overexpressing clones induced at 20 hr and examined at 48 hr APF. (E) A two-cell clone (faint GFP) is composed of two ACs (Cut^+^). (F) A three-cell clone (upper panel) contains one AC (Cut^+^); a two-cell clone (one with faint GFP and one with strong GFP; lower panel) is composed of two ACs (Cut^+^). (G) A three-cell clone is composed of one EC (strong GFP and distinct cellular morphology) and two ACs (Cut^+^). (H) Quantification of clone distribution according to clone composition. pIIa/pIIb: clones containing one pIIa and pIIb cell during division. pIIb/pIIb: clones containing two pIIb cells during division, which gives rise to two ACs at 48 hr APF. Fisher’s exact test was used (***P < 0.001). AC, apical cell; APF, after puparium formation; βGal, β-galactosidase; EC, epithelial cell; GFP, green fluorescent protein; Hnt, Hindsight; NICD, Notch intracellular domain; SC, secretory cell; Su(H), Suppressor of Hairless.

Due to the transient nature of pIIa/pIIb division, we have not been able to directly visualize Notch activity in pIIa/pIIb cells; however, we consistently detected faint expression of a reporter of Notch activity in ACs at 48 hr APF. This result suggests that pIIb cells might receive Notch signaling (Figure 1B and Sun and Spradling 2013). To further probe this possibility, we used a different Notch activity reporter and again observed faint Notch activity in ACs compared to SCs (Figure 5A), indicating a potential role of Notch signaling in pIIb specification. This finding is consistent with the observation that *NICD*-overexpressing clones induced at 24 hr APF occasionally consist of two cells with faint Hnt and GFP, presumably ACs (Figure 5B). We observed such clones more frequently when we induced them at 20 hr APF.

We next aimed to demonstrate that *NICD*-overexpressing clones with faint Hnt and GFP are indeed ACs. We examined the expression of Cut, which is specifically expressed in ACs at 48 hr APF (Figure 1B). When we induced the clones at 20 hr APF, 83% of control SC clones consisted of three cells with one Cut^+^ AC, indicating a flip-out event in SUPs (Figure 5, C and D). Interestingly, ACs always had the lowest level of GFP expression (Figure 5C; also see *Results* in later section). The remaining clones were composed of four or five cells, an AC, an SC, a BC, and one or two ECs, indicating a flip-out event in a gland precursor (P0; Figure 1A). We never observed two-cell clones with Cut^+^ cells (Figure 5D). In contrast, 49% of *NICD*-overexpressing clones were two-cell clones, all of which consisted of two Cut^+^ ACs (Figure 5, D and E), indicating a pIIa-to-pIIb transformation. Occasionally, a clone had one AC with low GFP and one AC with high GFP (Figure 5F); the one with low GFP likely resulted from the original pIIb cell (giving rise to the AC) and the one with high GFP likely resulted from an incomplete transformation of a pIIa to a pIIb cell. The three-cell clones with *NICD* overexpression consisted of either one AC and two SCs (Figure 5F and Figure S6A) or two ACs and one EC (Figure 5G and Figure S6B; note the distinct morphology and GFP intensity of the ECs). The latter clones were derived from gland precursors and had a pIIa-to-pIIb transformation. In total, 56% of *NICD*-overexpressing clones had a pIIa-to-pIIb transformation when induced at 20 hr APF (Figure 5H). These data suggest that Notch signaling is sufficient for pIIb fate determination in the secretory lineage.

### Notch signaling is required for pIIb fate determination

**Figure 6:**
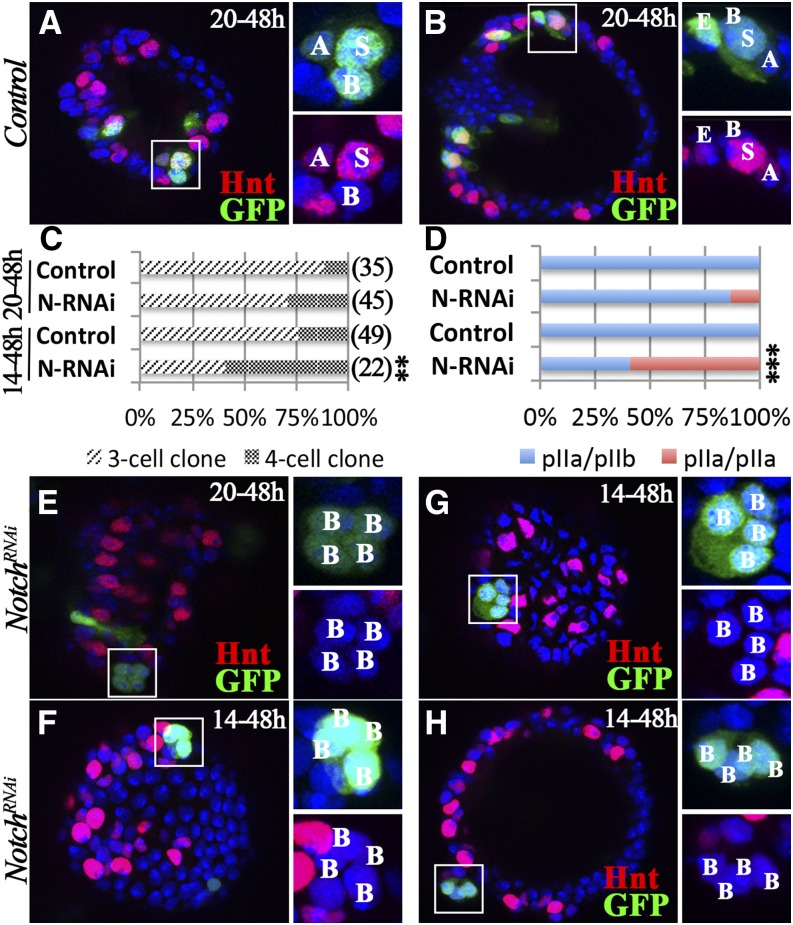
Notch signaling is required for pIIb fate. (A and B) Representative control clones induced at 20 hr and examined at 48 hr APF. A three-cell clone is shown in (A), and a four-cell clone is shown in (B) with clone composition labeled according to Hnt expression (red). (C and D) Quantification of clone distribution according to clone size (C) and composition (D). The Fisher’s exact test was used (**P < 0.01 and ***P < 0.001). (E) A representative *N*-knockdown clone induced at 20 hr APF shows four BC-like cells without Hnt expression (red) at 48 hr APF. (F–H) Representative *N*-knockdown clones induced at 14 hr and examined at 48 hr APF. (F) A four-cell clone with four BCs (same size). (G) A four-cell clone with four BCs (two small and two big). (H) A four-cell clone with four BCs detaching from the lumen. APF, after puparium formation; BC, basal cell; GFP, green fluorescent protein; Hnt, Hindsight; *N*, *Notch*; RNAi, RNA interference.

We used the same flip-out Gal4 system to investigate whether Notch signaling is required for pIIb fate determination. When induced at 20 hr APF, control clones were composed of 89% three-cell clones (derived from SUPs) and 11% four- or five-cell clones with one or two ECs (derived from P0; Figure 6, A–C). In contrast, 71% of *N*-knockdown clones were three-cell clones (Figure 6C), the majority of which consisted of one AC (faint Hnt) and two BCs (Hnt^−^). This is similar to the clones induced at 24 hr APF (Figure 4G) and further supports the role of Notch in SC specification. Among the rest of the four-cell clones, we observed four BC-like cells (Hnt-; Figure 6E), indicating a pIIb-to-pIIa transformation and a subsequent SC-to-BC transformation. In total, 14% of *N*-knockdown clones showed a pIIb-to-pIIa transformation (Figure 6D).

To test whether induction of clones at an earlier time point could increase the pIIb-to-pIIa transformation rate, we induced clones at 14 hr APF. This experimental scheme resulted in 76% three-cell clones (SUP clones) and 24% four- or five-cell clones (P0 clones) in the control group (Figure 6C). In contrast, we observed significantly more four-cell clones (59%) in the *N*-knockdown group (Figure 6C), all of which consisted of four BC-like cells without Hnt expression (Figure 6, F–H and File S1). Occasionally, two of the clone cells were slightly smaller than the other two (Figure 6G); this likely indicates an incomplete pIIb-to-pIIa transformation, as pIIb-derived ACs are smaller than SCs or BCs. Most strikingly, we observed one clone with eight BCs (File S2); this was likely a gland precursor clone that produced two SUPs and subsequently eight BCs. In total, we observed that 59% of *N*-knockdown clones had a pIIb-to-pIIa transformation (Figure 6D). We observed similar four-BC clones when *Su(H)^DN^* was overexpressed, although less frequently (Figure S7). These data suggest that Notch signaling is required for pIIb fate specification.

We note that *N*-knockdown clones were rarer when induced at 14 hr APF (22 clones from 47 *N^RNAi^* spermathecae *vs.* 39 clones from 14 control spermathecae). We frequently observed four-cell *N*-knockdown clones detaching away from the central lumen (Figure 6H), which might account for the clone loss. The clone detachment was not caused by the loss of SCs, because *N*-knockdown clones with two BCs were readily observed when induced at 24 hr APF (Figure 4, D and G); instead, we believe the detachment was caused by the loss of pIIb cells/ACs. Altogether, our data suggest that Notch signaling is required and sufficient for pIIb fate and that pIIb cells/ACs are required for securing the secretory unit to the epithelial lumen.

### Notch activation in LEPs and SCs utilizes different ligand mechanisms

**Figure 7:**
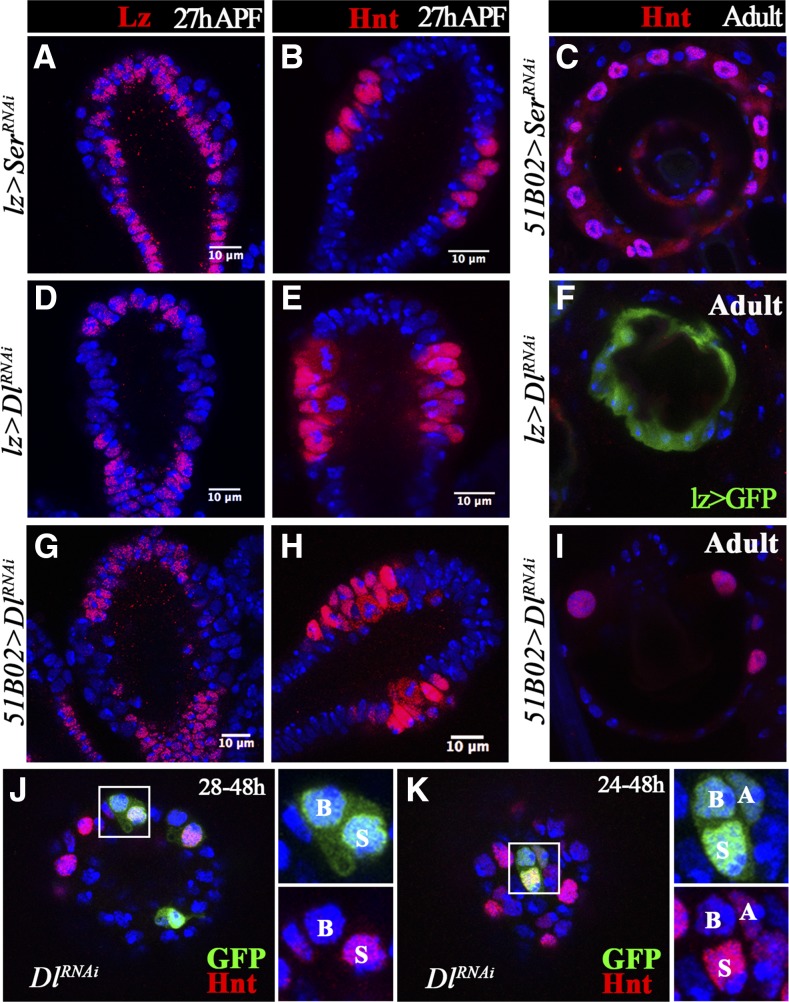
Dl, but not Ser, is required for LEP fate specification. (A and B) *Ser* knockdown with *lz-Gal4* shows normal Lz (A) and Hnt (B) expression in spermatheca at 27 hr APF. (C) Adult spermathecae with *Ser* knockdown in *51B02-Gal4*-expressing cells have normal secretory cells marked by Hnt expression. (D–I) *Dl* knockdown in *lz-Gal4*- (D–F) or *51B02-Gal4*-expressing cells (G–I) leads to loss of Lz (D and G) and gain of Hnt (E and H) expression at the middle region of spermathecal head at 27 hr APF. Few secretory cells were formed in adult spermathecae (F–I). (J–K) *Dl*-knockdown clones induced at 28 (J) or 24 hr (K) and examined at 48 hr APF. These clones have normal cell composition. Hnt expression (red) is used to marked the SCs. APF, after puparium formation; Dl, Notch ligand δ; Hnt, Hindsight; LEP, lumen epithelial precursor; Lz, Lozenge; RNAi, RNA interference; SC, secretory cell; Ser, Serrate.

In *Drosophila*, two Notch ligands, Dl and Ser, have been found to activate Notch signaling. To identify the ligand for Notch signaling in spermathecal lineage specification, we first knocked down S*er* with two different RNAi lines in all gland precursor cells with *lz-Gal4* or in the middle region of the spermathecal head with *51B02-Gal4*. Lz and Hnt were properly expressed in spermathecae at 27 hr APF, and SCs were properly formed in adult spermathecae (Figure 7, A–C), indicating that Ser is unlikely to be the ligand for Notch signaling in the spermathecal lineage. In contrast, *Dl*-knockdown spermathecae showed similar LEP-to-SUP transformation (loss of Lz and gain of Hnt expression) as *N*-knockdown spermathecae at 26 hr APF (Figure 7, D, E, G, and H). In addition, *Dl*-knockdown spermathecae had no SCs formed in the adult (Figure 7, F and I). All these data suggest that Dl, but not Ser, is the ligand for Notch signaling during LEP–SUP specification.

We also tried to determine whether Dl is the ligand for Notch signaling in later stages using the same flip-out clone system to knock down *Dl*. Unlike the *N*-knockdown clones, *Dl*-knockdown clones induced at 28 or 24 hr APF had normal clone composition at 48 hr APF (Figure 7, J and K; 48 and 42 clones examined, respectively). This suggests that SCs utilize a different mechanism from LEPs to activate Notch signaling, such as a ligand-independent mechanism. Due to technical challenges, we were unable to generate *Dl* or *Dl*/*Ser* double-null clones to determine whether Notch signaling in SCs, as well as pIIb cells, was activated ligand-independently or redundantly by Dl and Ser.

### Each cell type in the secretory lineage shows a distinct cellular morphology

**Figure 8:**
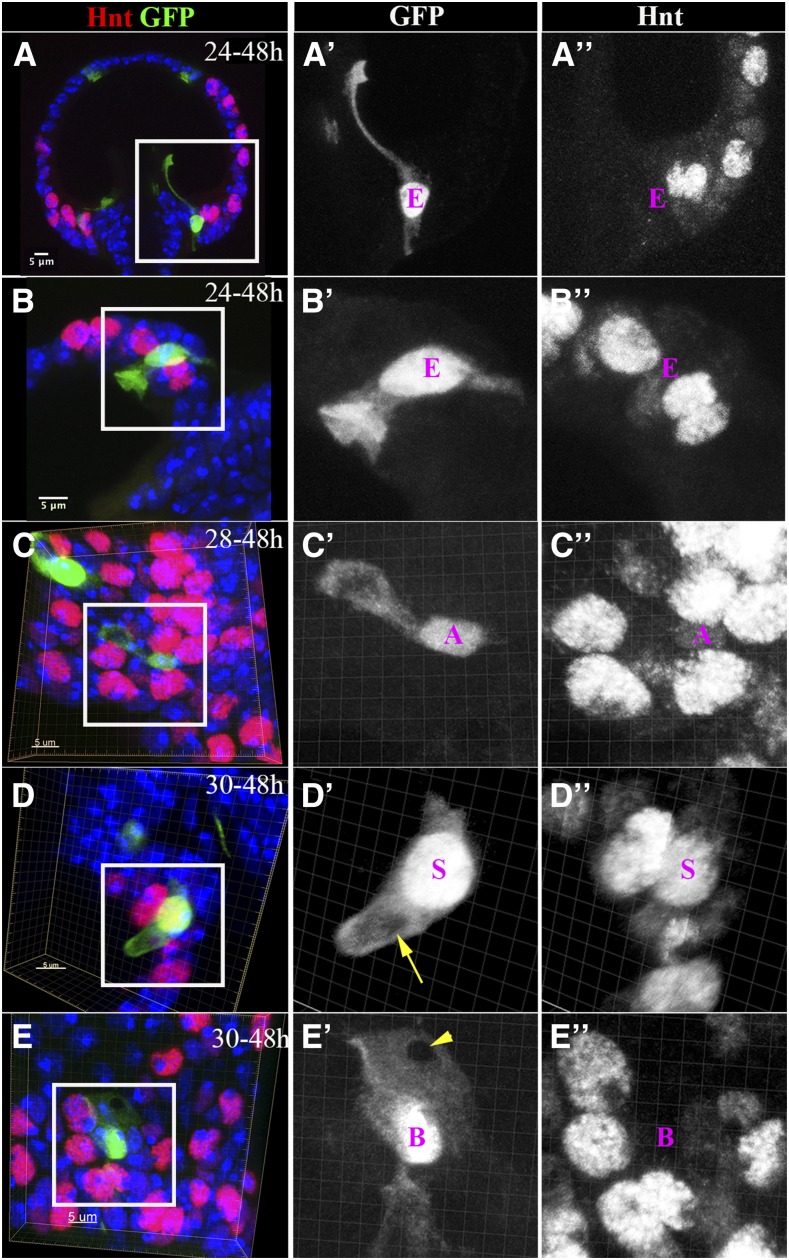
Cellular morphology of spermathecal lineage cells. (A and B) Single-EC clones induced at 24 hr and examined at 48 hr APF. (A) The EC localized at the junction of spermathecal lumen, and the duct has a long cytoplasmic protrusion in line with the introvert. (B) The EC localized in the middle region of the spermathecal head has an inverted umbrella-shaped apical membrane protruding into the lumen. The cytoplasm is marked by GFP [green in (A and B) and white in (A’–B’)]. (C) A single-AC clone shows the AC with an apical cytoplasmic bulge (C’). The AC is recognizable by the faint Hnt expression (white in C”). (D) A single-SC clone shows a finger-like apical protrusion with a hole in the middle (yellow arrow in D’). The SC is marked by strong Hnt expression (white in D”). (E) A single-BC clone shows a mesh-like apical membrane with a hole in the apical tip (arrowhead in E’). The BC is recognizable by the absence of Hnt expression (white in E”). The images in (B–E) are generated from three-dimensional volume rendering. AC, apical cell; BC, basal cell; EC, epithelial cell; GFP, green fluorescent protein; Hnt, Hindsight; SC, secretory cell.

Cells fulfilling different biological functions frequently acquire different cellular morphology. Indeed, we observed that each cell in the spermathecal lineage was morphologically distinct when we generated single-cell clones using the flip-out Gal4 system. LEP-derived ECs typically had the highest GFP expression and lacked Hnt expression (Figure 6B and Figure 8, A and B). ECs close to the spermathecal introvert (the portion of the spermathecal duct opening that protrudes into the spermathecal lumen) (Pitnick *et al.* 1999) had a thin, elongated apical protrusion that aligned to the introvert at 48 hr APF (Figure 8A); this may be involved in introvert formation. In contrast, ECs along the spermathecal lumen had an inverted umbrella-shaped apical membrane protruding into the lumen (Figure 8B); this may be involved in lumen cuticle formation, which is supported by our finding that the transformation of LEPs into SUPs caused by Notch signaling disruption leads to a smaller spermathecal lumen (Figure 3, G–H).

The three cells in the secretory unit were reported to wrap around each other, forming a concentric ring (Mayhew and Merritt 2013). To distinguish each cell’s morphology in this unit, we induced single-AC clones at 28 hr APF and examined morphology at 48 hr APF. In contrast to ECs, ACs had lower GFP expression, smaller cell bodies, and faint Hnt expression (Figure 4F, Figure 6, A and B, and Figure 8C). Most strikingly, ACs had a bulge-like apical protrusion connected to the main cellular body with a thin cytoplasmic tubule (Figure 8C’). This bulge-like apical protrusion may facilitate attachment of ACs to the epithelial lumen, thus securing the three-cell secretory unit to the lumen. Both SCs and BCs had a medium level of GFP expression. Whereas SCs had the biggest nuclei and a high level of Hnt expression, BCs had no Hnt expression (Figure 8, D and E). In addition, SCs had a finger-like apical protrusion with a hole in the middle (Figure 8D), where the end-apparatus is likely to form. These finger-like apical membranes presumably form microvilli to surround the end-apparatus in the adult. In contrast, BCs had a mesh-like apical membrane with a hole in the apical tip (Figure 8E). The role of BCs in the secretory unit is currently unknown, but these cells likely function to separate each secretory unit from one other. The distinct morphologies of ACs, BCs, and SCs comprise the sophisticated secretory unit with an end-apparatus and canal in adult spermathecae.

## Discussion

### Notch signaling and binary cell fate determination in class-III secretory glands

Through lineage tracing, we have previously determined the *Drosophila* spermathecal lineage with single-cell resolution (Figure 1A); however, it is unknown how each cell in this lineage is specified. In addition, the molecular mechanisms involved in other class-III secretory unit formations are unknown, despite their essential roles for insect physiology and behavior, including reproduction, digestion, defensive behavior, and social communication (Sreng 2006; Billen 2011; Giglio *et al.* 2011; Gomez‐Diaz and Benton 2013). Inspired by the enormous body of work on Notch in binary cell fate determination in the *Drosophila* sensory lineage (Lai and Orgogozo 2004; Schweisguth 2015), we investigated its role in the spermathecal lineage. Not surprisingly, Notch signaling is activated sequentially at each division in the spermathecal lineage and specifies LEPs (*vs.* SUPs), pIIb (*vs.* pIIa), and SCs (*vs.* BCs). This likely occurs in parovaria, as we frequently observed a similar cell fate transformation in parovaria when we manipulated Notch signaling. In contrast, it is surprising to us that Notch activation in LEPs and SCs apparently utilizes different ligand mechanisms, as knocking down *Dl* using the same RNAi construct is able to block LEP specification but not SC specification. Since *Ser* knockdown is also not required for SC specification, this may indicate that SCs utilize a ligand-independent mechanism to activate Notch signaling (Palmer and Deng 2015). Alternatively, both Dl and Ser may play redundant roles in activating Notch signaling. Due to technical challenges in generating mosaic clones, we were unable to definitively solve this question.

It is still unknown how the asymmetric cell fate and Notch signaling are established in the spermathecal lineage and whether the same machinery for asymmetry is involved in the spermathecal lineage as in the sensory lineage, such as Numb and Neuralized (Schweisguth 2015). The fact that ACs, BCs, and SCs have different cell size may also indicate the involvement of asymmetric cytokinesis in this lineage. The size difference also seems influenced by Notch signaling, similar to its role in asymmetric cleavage in neural precursor cells (Bhat 2014). Future work will be required to understand what determines the asymmetry and where the ligand source is for Notch signaling. It is interesting to note that Notch signaling is only observed at 26 hr APF in the middle region of the spermathecal head, where the double layer and secretory unit forms. Previous work has shown that precursor cells divide either perpendicularly or parallel to the lumen before 24 hr APF (Mayhew and Merritt 2013). Presumably, the perpendicular division leads to the double layer formation. Thus, the division axis may be one of the mechanisms to control asymmetric Notch signaling during LEP–SUP fate determination.

### The same Notch signaling yet the opposite outcome in the spermathecal lineage

The Notch pathway is evolutionarily conserved, plays pleiotropic roles in multiple organ systems, and frequently results in completely opposite biological consequences in different organs or different cell lineages within the same tissue (Valdez and Xin 2013). It is quite striking that the same Notch signaling inhibits Hnt expression in LEPs but activates Hnt expression in SCs, which is only two divisions away from LEPs. Positive regulation of Hnt by Notch signaling has also been reported in ovarian follicle cells and the hematopoietic lineage, and Hnt is likely the direct target of Su(H) (Sun and Deng 2007; Terriente-Felix *et al.* 2013). It is unclear why Notch signaling in LEPs cannot activate Hnt. Likewise, Notch signaling inhibits Cut expression in LEPs but activates Cut expression in pIIb/ACs. The positive and negative regulation of Cut by Notch signaling has been found in wing imaginal discs (Neumann and Cohen 1996; Jia *et al.* 2016) and ovarian follicle cells (Sun and Deng 2005), respectively, but these opposite effects of Notch on Cut expression have never been observed in the same lineage. Thus, the spermathecal lineage provides an extreme example of Notch signaling having opposite biological outcomes in different cellular environments. It is not known whether the different ligand mechanism for Notch activation in LEPs, SCs, and possibly pIIb, is the cause of the different outcome of the same Notch signaling. Further investigation into the molecular mechanisms of Notch regulation of Hnt and Cut expression in the spermathecal lineage will shed light on this fundamental question.

### Role of each cell in the spermathecal lineage for secretory unit formation

In comparison to class-I and class-II SCs, class-III SCs have the most complicated structure and discharge their secretion through a cuticular end-apparatus and canal (Noirot and Quennedey 1974). Most of the early work has been centered in ultrastructure characterization of these SCs/secretory units in a variety of insect glands using electron microscopes (Quennedey 1998). However, the formation of such sophisticated units is only minimally understood in cockroach tergal glands, in which the precursor divides to form a four-cell unit, including an envelope cell, an SC, a canal cell, and a ciliary cell (Sreng and Quennedey 1976). Both the ciliary cell and the envelope cell disappear in the adult gland through apoptosis regulated by a brain factor (Sreng 1998). It is speculated that the ciliary cell functions as a guide in the center, whereas the SC, canal cell, and envelope cell wrap around the ciliary cell to form a concentric ring. This hypothesis is solely based on ultrastructural morphology and has never been genetically tested.

In contrast to cockroach tergal glands, secretory units of *Drosophila* spermathecae are built through a three-cell unit, including an AC, a BC, and an SC. No cilium is involved in the formation of this secretory unit (Sun and Spradling 2012), but the three cells wrap around each other to form a concentric ring (Mayhew and Merritt 2013) and the AC and the BC disappear in the adult glands (Sun and Spradling 2012). It remains largely unknown what each cell’s contribution is in building the adult secretory unit. By manipulating Notch signaling, we observed that the transformation of LEPs into SUPs, or of the pIIb cell to a pIIa cell, leads to detachment of the secretory unit from the epithelial lumen, indicating an essential role of ECs and ACs in securing secretory units to the lumen. In addition, the AC sends an elongated cytoplasmic protrusion into the lumen with a bulge at the end, whereas ECs form the inverted umbrella-shaped apical process. Thus, we propose that ACs are molded into the epithelial lumen because of their apical protrusion and bulge, providing a guide around which the SC and BC can wrap. Without ACs, BCs and SCs would have no attachment and would get lost. Similarly, without ECs, ACs could not be held to the epithelial lumen, and the entire three-cell unit would get lost. In this sense, the function of ACs is equivalent to ciliary cells in cockroach tergal glands. With a better understanding of each cell type and the essential transcription factors for each cell fate, we will be able to precisely interrogate the function of each cell in the secretory unit and generally better understand class-III secretory unit formation. This work will also generate tools to precisely manipulate gland secretions and decipher their physiological functions.

## Supplementary Material

Supplemental material is available online at www.g3journal.org/lookup/suppl/doi:10.1534/g3.117.040212/-/DC1.

## References

[bib1] Allen A. K. Spradling A. C., 2008 The *Sf1*-related nuclear hormone receptor *Hr39* regulates *Drosophila* female reproductive tract development and function. Development 135: 311–321. 18077584 10.1242/dev.015156

[bib2] Anderson R. C., 1945 A study of the factors affecting fertility of lozenge females of Drosophila melanogaster. Genetics 30: 280–296. 17247158 10.1093/genetics/30.3.280 PMC1209287

[bib3] Artavanis-Tsakonas S. Rand M. D. Lake R. J., 1999 Notch signaling: cell fate control and signal integration in development. Science 284: 770–776. 10221902 10.1126/science.284.5415.770

[bib4] Bhat K. M., 2014 Notch signaling acts before cell division to promote asymmetric cleavage and cell fate of neural precursor cells. Sci. Signal. 7: ra101. 25336614 10.1126/scisignal.2005317

[bib5] Billen J., 2011 Exocrine glands and their key function in the communication system of social insects. Formos. Entomol 31: 75–84.

[bib6] Blochlinger K. Jan L. Y. Jan Y. N., 1991 Transformation of sensory organ identity by ectopic expression of Cut in Drosophila. Genes Dev. 5: 1124–1135. 1676691 10.1101/gad.5.7.1124

[bib7] Cattenoz P. B. Delaporte C. Bazzi W. Giangrande A., 2016 An evolutionary conserved interaction between the Gcm transcription factor and the SF1 nuclear receptor in the female reproductive system. Sci. Rep. 6: 37792. 27886257 10.1038/srep37792 PMC5122895

[bib8] Chatterjee S. S. Uppendahl L. D. Chowdhury M. A. Ip P. L. Siegal M. L., 2011 The female-specific Doublesex isoform regulates pleiotropic transcription factors to pattern genital development in *Drosophila*. Development 138: 1099–1109. 21343364 10.1242/dev.055731

[bib50] Crew J. R. Batterham P. Pollock J. A., 1997 Developing compound eye in lozenge mutants of Drosophila: lozenge expression in the R7 equivalence group. Dev. Gene. Evol. 206: 481–493. 10.1007/s004270050079 27747375

[bib9] Dallai R. Zizzari Z. V. Fanciulli P. P., 2008 Fine structure of the spermatheca and of the accessory glands in *Orchesella villosa* (Collembola, Hexapoda). J. Morphol. 269: 464–478. 18157861 10.1002/jmor.10595

[bib10] de Celis J. F. Bray S., 1997 Feed-back mechanisms affecting Notch activation at the dorsoventral boundary in the *Drosophila* wing. Development 124: 3241–3251. 9310319 10.1242/dev.124.17.3241

[bib11] Eberhard W. G., 1996 *Female Control: Sexual Selection by Cryptic Female Choice*. Princeton University Press, Princeton, NJ.

[bib12] Filosi M. Perotti M. E., 1975 Fine structure of the spermatheca of Drosophila melanogaster Meig. J. Submicrosc. Cytol. 7: 259–270.

[bib13] Furriols M. Bray S., 2001 A model Notch response element detects suppressor of Hairless–dependent molecular switch. Curr. Biol. 11: 60–64. 11166182 10.1016/s0960-9822(00)00044-0

[bib14] Giglio A. Brandmayr P. Talarico F. Brandmayr T. Z., 2011 Current knowledge on exocrine glands in carabid beetles: structure, function and chemical compounds. ZooKeys 100: 193–201. 10.3897/zookeys.100.1527 PMC3131016 21738412

[bib15] Gomez‐Diaz C. Benton R., 2013 The joy of sex pheromones. EMBO Rep. 14: 874–883. 24030282 10.1038/embor.2013.140 PMC3807217

[bib16] Gupta B. L. Smith D. S., 1969 Fine structural organization of the spermatheca in the cockroach, *Periplaneta americana*. Tissue Cell 1: 295–324. 18631470 10.1016/s0040-8166(69)80027-3

[bib17] Happ G. M. Happ C. M., 1977 Cytodifferentiation in the accessory glands of *Tenebrio molitor*. III. Fine structure of the spermathecal accessory gland in the pupa. Tissue Cell 9: 711–732. 610008 10.1016/0040-8166(77)90037-4

[bib18] Jia D. Bryant J. Jevitt A. Calvin G. Deng W.-M., 2016 The ecdysone and Notch pathways synergistically regulate cut at the dorsal–ventral boundary in *Drosophila* wing discs. J. Genet. Genomics 43: 179–186. 27117286 10.1016/j.jgg.2016.03.002 PMC5391978

[bib19] Keisman E. L. Christiansen A. E. Baker B. S., 2001 The sex determination gene *doublesex* regulates the A/P organizer to direct sex-specific patterns of growth in the *Drosophila* genital imaginal disc. Dev. Cell 1: 215–225. 11702781 10.1016/s1534-5807(01)00027-2

[bib20] Laghezza Masci V. Di Luca M. Gambellini G. Taddei A. R. Belardinelli M. C., 2015 Reproductive biology in Anophelinae mosquitoes (Diptera, Culicidae): fine structure of the female accessory gland. Arthropod Struct. Dev. 44: 378–387. 25895726 10.1016/j.asd.2015.04.003

[bib21] Lai E. C. Orgogozo V., 2004 A hidden program in *Drosophila* peripheral neurogenesis revealed: fundamental principles underlying sensory organ diversity. Dev. Biol. 269: 1–17. 15081353 10.1016/j.ydbio.2004.01.032

[bib22] Lococo D. Huebner E., 1980 The development of the female accessory gland in the insect *Rhodnius prolixus*. Tissue Cell 12: 795–813. 7010677 10.1016/0040-8166(80)90030-0

[bib23] Manier M. K. Belote J. M. Berben K. S. Novikov D. Stuart W. T., 2010 Resolving mechanisms of competitive fertilization success in *Drosophila melanogaster*. Science 328: 354–357. 20299550 10.1126/science.1187096

[bib24] Mayhew M. L. Merritt D. J., 2013 The morphogenesis of spermathecae and spermathecal glands in *Drosophila melanogaster*. Arthropod Struct. Dev. 42: 385–393. 23872109 10.1016/j.asd.2013.07.002

[bib25] Mukherjee T. Kim W. S. Mandal L. Banerjee U., 2011 Interaction between Notch and Hif-alpha in development and survival of *Drosophila* blood cells. Science 332: 1210–1213. 21636775 10.1126/science.1199643 PMC4412745

[bib26] Neumann C. J. Cohen S. M., 1996 A hierarchy of cross-regulation involving Notch, wingless, vestigial and cut organizes the dorsal/ventral axis of the Drosophila wing. Development 122: 3477–3485. 8951063 10.1242/dev.122.11.3477

[bib27] Noirot C. Quennedey A., 1974 Fine structure of insect epidermal glands. Annu. Rev. Entomol. 19: 61–80.

[bib28] Palmer W. H. Deng W.-M., 2015 Ligand-Independent mechanisms of Notch activity. Trends Cell Biol. 25: 697–707. 26437585 10.1016/j.tcb.2015.07.010 PMC4628868

[bib29] Pascini T. V. Ramalho-Ortigão M. Martins G. F., 2012 Morphological and morphometrical assessment of spermathecae of *Aedes aegypti* females. Mem. Inst. Oswaldo Cruz 107: 705–712. 22990957 10.1590/s0074-02762012000600001

[bib30] Pascini T. V. Ramalho-Ortigão J. M. Martins G. F., 2013 The fine structure of the spermatheca in *Anopheles aquasalis* (Diptera: Culicidae). Ann. Entomol. Soc. Am. 106: 857–867.

[bib31] Pignoni F. Zipursky S. L., 1997 Induction of Drosophila eye development by decapentaplegic. Development 124: 271–278. 9053304 10.1242/dev.124.2.271

[bib32] Pitnick S. Markow T. Spicer G. S., 1999 Evolution of multiple kinds of female sperm-storage organs in *Drosophila*. Evolution 53: 1804. 10.1111/j.1558-5646.1999.tb04564.x 28565462

[bib33] Pitsouli C. Perrimon N., 2013 The homeobox transcription factor Cut coordinates patterning and growth during *Drosophila* airway remodeling. Sci. Signal. 6: ra12. 23423438 10.1126/scisignal.2003424 PMC3982146

[bib34] Prokupek A. Hoffmann F. Eyun S. Moriyama E. Zhou M., 2008 An evolutionary expressed sequence tag analysis of Drosophila spermatheca genes. Evolution 62: 2936–2947. 18752616 10.1111/j.1558-5646.2008.00493.x

[bib35] Prokupek A. M. Kachman S. D. Ladunga I. Harshman L. G., 2009 Transcriptional profiling of the sperm storage organs of *Drosophila melanogaster*. Insect Mol. Biol. 18: 465–475. 19453766 10.1111/j.1365-2583.2009.00887.x

[bib36] Quennedey A., 1998 Insect epidermal gland cells: ultrastructure and morphogenesis, pp. 177–207 in *Microscopic Anatomy of Invertebrates*, edited by Harrison F. W. Wiley-Liss, London.

[bib37] Schnakenberg S. L. Matias W. R. Siegal M. L., 2011 Sperm-storage defects and live birth in *Drosophila* females lacking spermathecal secretory cells. PLoS Biol. 9: e1001192. 22087073 10.1371/journal.pbio.1001192 PMC3210755

[bib38] Schweisguth F., 2015 Asymmetric cell division in the *Drosophila* bristle lineage: from the polarization of sensory organ precursor cells to Notch-mediated binary fate decision. Wiley Interdiscip. Rev. Dev. Biol. 4: 299–309. 25619594 10.1002/wdev.175 PMC4671255

[bib39] Shaw W. R. Teodori E. Mitchell S. N. Baldini F. Gabrieli P., 2014 Mating activates the heme peroxidase HPX15 in the sperm storage organ to ensure fertility in *Anopheles gambiae*. Proc. Natl. Acad. Sci. USA 111: 5854–5859. 24711401 10.1073/pnas.1401715111 PMC4000814

[bib40] Sreng L., 1998 Apoptosis-inducing brain factors in maturation of an insect sex pheromone gland during differentiation. Differentiation 63: 53–58.

[bib41] Sreng L., 2006 Cockroach tergal glands producing female sex attractant pheromones and male aphrodisiacs in particular in the subfamily Blaberinae (Blattaria: Blaberidae). Eur. J. Entomol. 103: 817–829.

[bib42] Sreng L. Quennedey A., 1976 Role of a temporary ciliary structure in the morphogenesis of insect glands. An electron microscope study of the tergal glands of male *Blattella germanica* L. (Dictyoptera, Blattellidae). J. Ultrastruct. Res. 56: 78–95. 948103 10.1016/s0022-5320(76)80142-6

[bib43] Sun J. Deng W.-M., 2005 Notch-dependent downregulation of the homeodomain gene cut is required for the mitotic cycle/endocycle switch and cell differentiation in *Drosophila* follicle cells. Development 132: 4299–4308. 16141223 10.1242/dev.02015 PMC3891799

[bib44] Sun J. Deng W.-M., 2007 Hindsight mediates the role of Notch in suppressing hedgehog signaling and cell proliferation. Dev. Cell 12: 431–442. 17336908 10.1016/j.devcel.2007.02.003 PMC1851662

[bib45] Sun J. Spradling A. C., 2012 NR5A nuclear receptor Hr39 controls three-cell secretory unit formation in *Drosophila* female reproductive glands. Curr. Biol. 22: 862–871. 22560612 10.1016/j.cub.2012.03.059 PMC3397175

[bib46] Sun J. Spradling A. C., 2013 Ovulation in *Drosophila* is controlled by secretory cells of the female reproductive tract. Elife 2: e00415. 23599892 10.7554/eLife.00415 PMC3628084

[bib47] Terriente-Felix A. Li J. Collins S. Mulligan A. Reekie I., 2013 Notch cooperates with Lozenge/Runx to lock haemocytes into a differentiation programme. Development 140: 926–937. 23325760 10.1242/dev.086785 PMC3557782

[bib48] Valdez J. M. Xin L., 2013 The dual nature of Notch in tissue homeostasis and carcinogenesis. Cell Cycle 12: 541. 23370389 10.4161/cc.23671 PMC3594250

[bib49] Zeng X. Chauhan C. Hou S. X., 2010 Characterization of midgut stem cell– and enteroblast-specific Gal4 lines in Drosophila. Genesis 48: 607. 20681020 10.1002/dvg.20661 PMC2958251

