# Supplementary figures and images for: Dynamic Notch Signaling Specifies Each Cell Fate in Drosophila Spermathecal Lineage
Source: G3 (Bethesda). 2017 Mar 3;7(5):1417–27. doi: 10.1534/g3.117.040212 (PMC5427495; doi:10.1534/g3.117.040212)

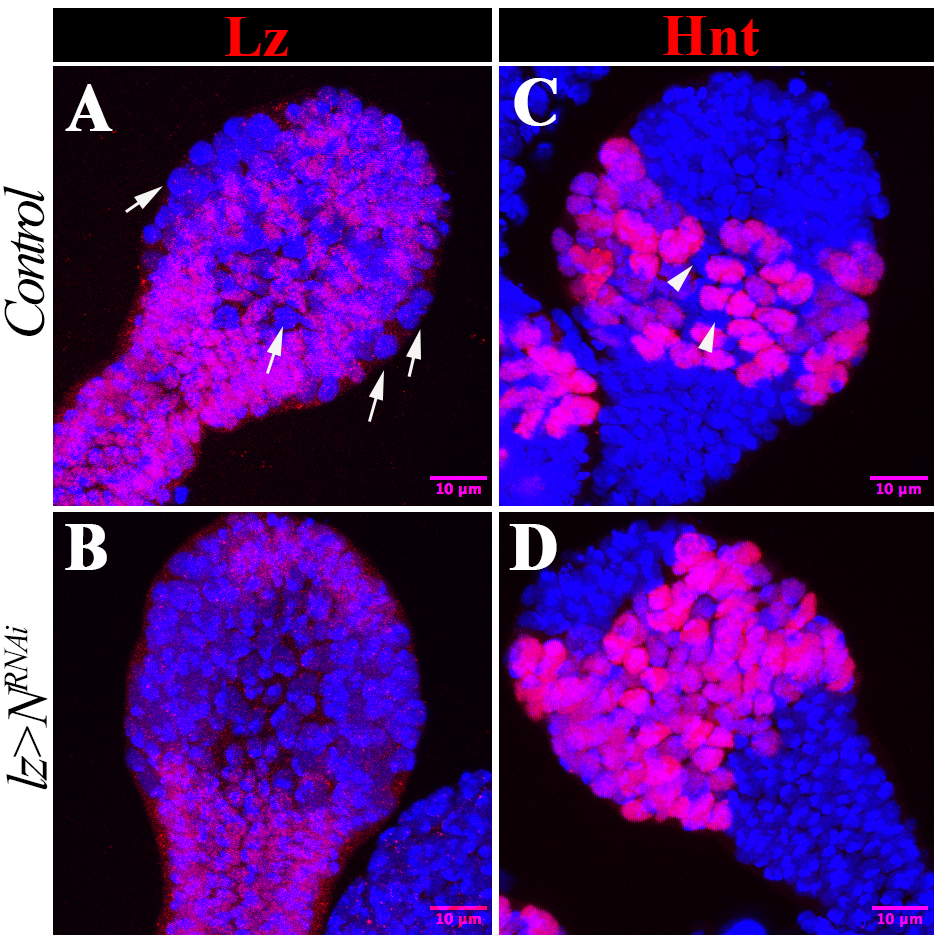

Supplement: Supplementary file 1 [file 1417FigureS1.tif]

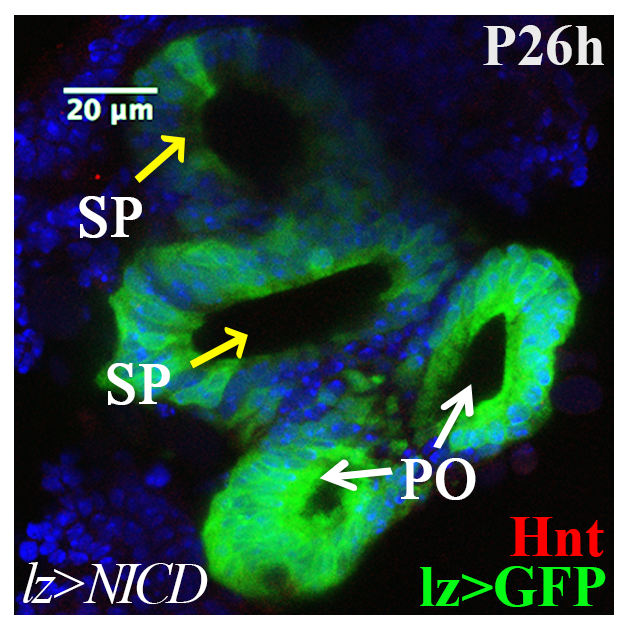

Supplement: Supplementary file 2 [file 1417FigureS2.tif]

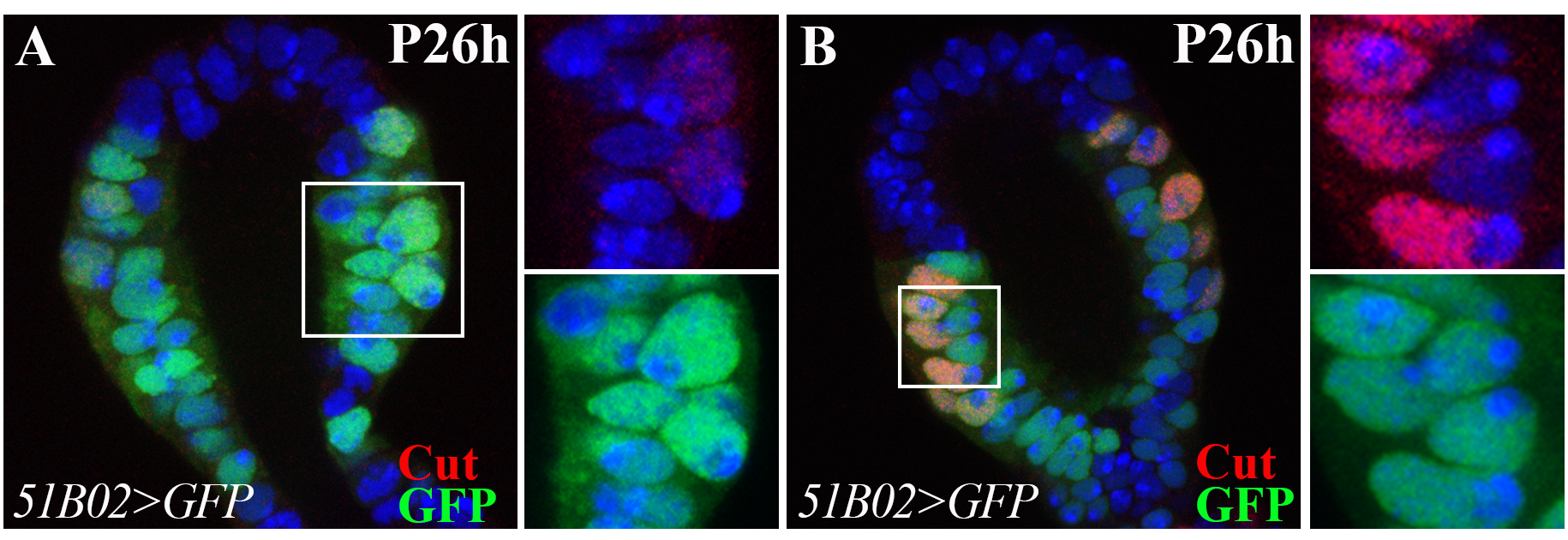

Supplement: Supplementary file 3 [file 1417FigureS3.tif]

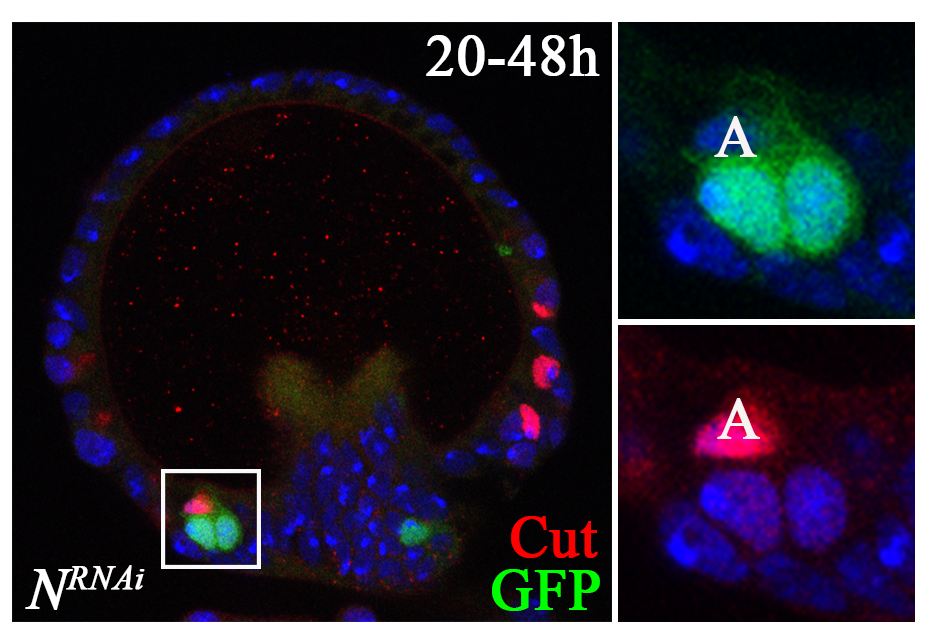

Supplement: Supplementary file 4 [file 1417FigureS4.tif]

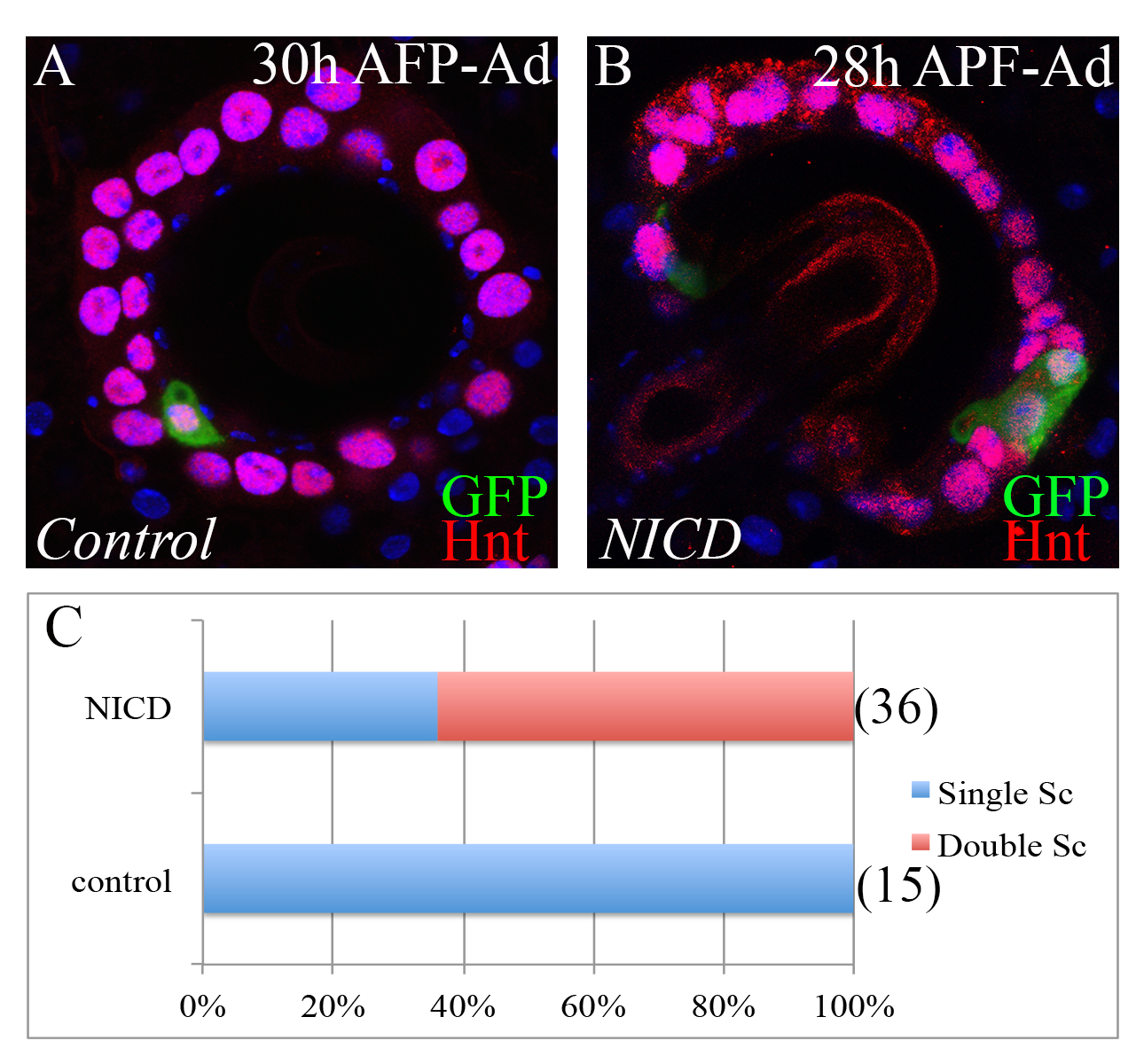

Supplement: Supplementary file 5 [file 1417FigureS5.tif]

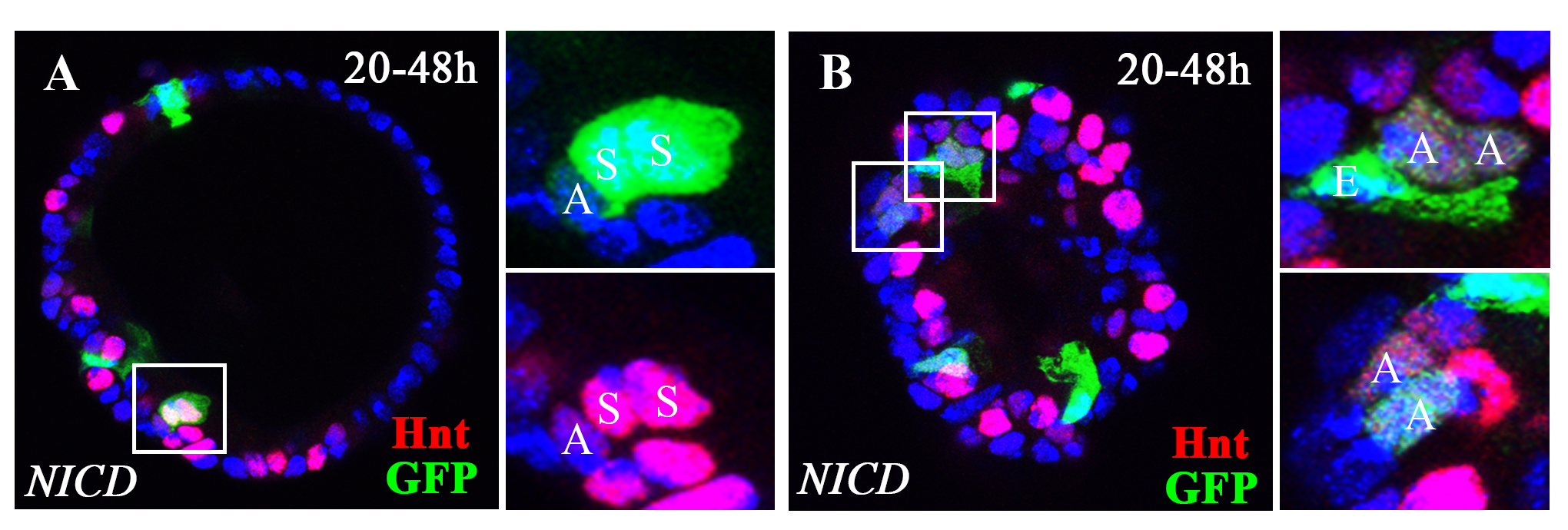

Supplement: Supplementary file 6 [file 1417FigureS6.tif]

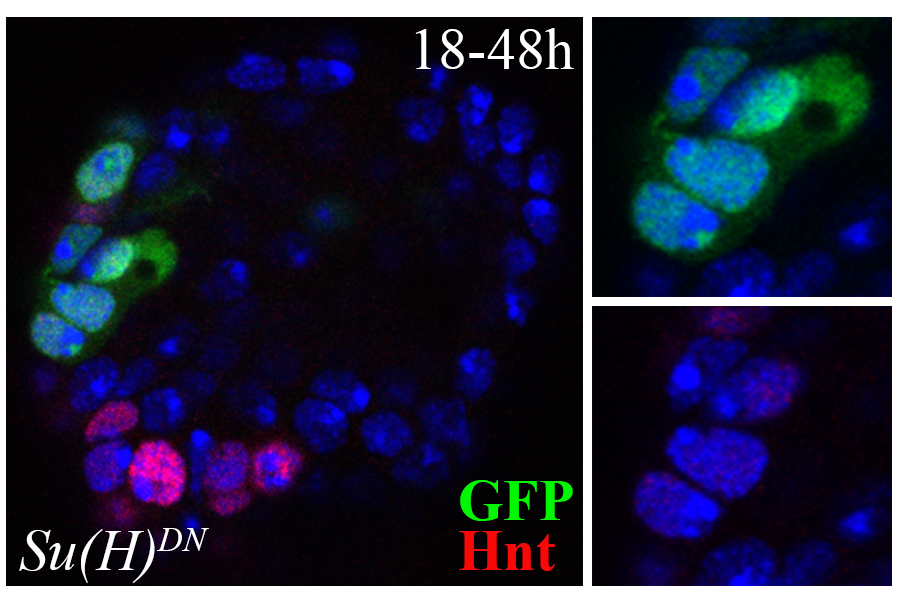

Supplement: Supplementary file 7 [file 1417FigureS7.tif]
