# Supplementary material for: Dynamic Notch Signaling Specifies Each Cell Fate in Drosophila Spermathecal Lineage
Source: G3 (Bethesda). 2017 Mar 3;7(5):1417–27. doi: 10.1534/g3.117.040212 (PMC5427495; doi:10.1534/g3.117.040212)
Supplement: Supplementary file 10 [file 1417FileS3.docx]

**Figure S1. Loss of Notch signaling leads to LEP-to-SUP transformation.** Images are generated with maximal Z projection from optical sections covering half of the spermathecal head. Expression of Lz (A-B) and Hnt (C-D) is shown in control (A and C) and *N*-knockdown (B and D) spermathecae at 27-28h APF. Arrows point to SUPs and arrowheads point to LEPs. Note the complete loss of Lz expression and gain of Hnt expression in the middle region of spermathecal head.

**Figure S2. Overexpression of *NICD* in gland precursor blocks spermatheca morphogenesis during early pupae development.** A genital imaginal disc with *lz-Gal4* driving *UAS-NICD* overexpression examined at 26h APF. The precursors of spermathecae (SP; pointed by yellow arrows) and parovaria (PO; pointed by white arrow) did not protrude out and the morphology resembles the structure at 14h APF (shown in (Sun and Spradling 2012). Gland precursor is marked by *lz-Gal4* driving *UAS-GFP* expression (green). Hnt expression was not detected (red; marking SUP cells).

**Figure S3. The expression pattern of *51B02-Gal4* in spermathecae.** (A-B) *51B02-Gal4* driving *UAS-GFP* expression (*51B02>GFP*; shown in green) in cells of both the apical and basal layers at the middle region of the spermathecal head at 26h APF. *51B02-Gal4* expression occurs at a slightly earlier time point than Cut expression (A).

**Figure S4. Cut expression in *N*-knockdown clones.** A representative *N*-knockdown clone induced at 20h and examined at 48h APF. The three-cell clone shows one AC with Cut expression (red).

**Figure S5. *NICD*-overexpressing clones produce two SCs in adult spermathecae.** (A-B) Representative control (A) and *NICD*-overexpressing (B) clones induced at 24h APF and examined in adult spermathecae. The control clone has a single SC (A) and the NICD-overexpressing clone has two SCs (B) in adult spermathecae. (C) Quantification of clone distribution in adult spermathecae, according to the number of SCs in each clone. The clones were induced at 24h APF.

**Figure S6. Expression of Hnt in *NICD*-overexpressing clones.** (A-B) Representative NICD-overexpressing clone induced at 20h and examined at 48h APF. Hnt expression is shown in red. (A) A three-cell clone composed of one AC (faint Hnt) and two SCs (strong Hnt). (B) A three-cell clone (upper panel) composed of one EC (no Hnt) and two ACs, and a two-cell clone (lower panel) composed of two ACs (faint Hnt).

**Figure S7. A representative *Su(H)^DN^*-overexpressing clone with four BCs.** The clone is induced at 18h and examined at 48h APF. Hnt expression (red) is not detected in the clone cells.

**File S1**. **A serial optical sectioning showing an *N*-knockdown clone with four BCs**. The clone was induced at 14h and examined at 48h APF.

**File S2. A serial optical sectioning showing an *N*-knockdown clone with eight BCs.** The clone was induced at 14h and examined at 48h APF.
